# A Novel Optimization Model and Application of Optimal Formula Design for Cu_x_Co_1−x_Fe_2_O_4_ Spinel-Based Coating Slurry in Relation to Near and Middle Infrared Radiation Strengthening

**DOI:** 10.3390/ma13102332

**Published:** 2020-05-19

**Authors:** Haiqing Du, Haifei An, Jian Zhang, Yuhao Ding, Chao Lian, Hao Bai

**Affiliations:** 1 Department of Mechanical Engineering, Zhejiang Industry Polytechnic College, Shaoxing 312000, China; 2 State Key Laboratory of Advanced Metallurgy, University of Science and Technology Beijing, Beijing 100083, China; b20190115@xs.ustb.edu.cn (H.A.); zhangjian606589@126.com (J.Z.); ustb2012dyh@163.com (Y.D.); lianchao@caep.org.cn (C.L.); baihao@metall.ustb.edu.cn (H.B.); 3 School of Metallurgical and Ecological Engineering, University of Science and Technology Beijing, Beijing 100083, China

**Keywords:** coating, infrared radiation, solid solution, optimization, emissivity

## Abstract

Coating slurry, in which the infrared radiation material is the main content, is applied in industrial furnaces to improve heat transfer and raise efficiency of furnaces. In this study, a ${\mathrm{Cu}}_{x}{\mathrm{Co}}_{1-x}{\mathrm{Fe}}_{2}O_{4}$ series material with a spinel structure was prepared, and the emissivity of different formulas in two wavebands (3–5 μm and 8–14 μm) was measured. To ensure that the material delivered high emissivity, optimization models were proposed using Matlab software, and proportions of CuO, Co_2_O_3_ and Fe_2_O_3_ were found to be 16.98%, 16.73% and 66.29%, respectively, in the optimal formula. Thus, using the ${\mathrm{Cu}}_{x}{\mathrm{Co}}_{1-x}{\mathrm{Fe}}_{2}O_{4}$ series material and additives, according to mixture regression method, fifteen formulas of coating slurry were designed, prepared and the emissivities were measured. With the Matlab software optimization model, the content of coating slurry was optimized and the corresponding emissivities were measured to be 0.931 and 0.905 in two wavebands, which is in agreement with the optimized calculation.

## 1. Introduction

Approximately 70% of the energy consumed in China is used in industries, and 25–40% of this energy is used in combustion within industrial furnaces [1]. However, the energy utilization efficiency of furnaces is generally low, and it is therefore important to take measures to improve their performance [2,3,4]. Currently, many technologies have been proposed to raise the efficiency of industrial furnaces, and it has been found that the enhancement of heat transfer in the heating process within furnaces is an efficient way of improving the furnaces’ efficiency [5]. In this respect, an infrared radiation coating was developed and applied to the surface of the inner walls of furnaces, and this is now widely used in industry [6]. The infrared radiation coating enhances the emissivity of the furnaces’ inner wall, and the radiation transfer is thus strengthened between the heated materials and the furnaces’ wall [7]. According to previous industrial practices, the application of infrared radiation coating can raise the efficiency by 5–30% [8].

The energy-saving mechanism of this coating has been the subject of considerable research, and a number of conclusions have been obtained: (a) the coating can improve the emissivity of the wall surface and thus strengthen the radiation heat transfer by absorbing more heat from the incident radiation and by reflecting less [9,10,11]; (b) the coating absorbs the incoming radiation from combustion gases, which have an intermittent spectral distribution, and it then radiates out continuous spectral radiation. It thus enables the heated material to absorb more heat by radiation [12,13,14,15]; (c) the wavelength of the high-temperature radiation within the furnaces is of 1–5 μm, and the coating can improve emissivity in this waveband [16,17,18].

Based on the experimental results, it has been found that ‘doping’ improves emissivity, as it can change the original regular crystalline structure and enable different kinds of ions to occupy the lattice site [19]. For instance, Ying Zhang et al. [20], introduced the RE (RE = La, Ce, Pr, Nd, Sm, Eu, Gd, Tb, and Dy) and Mn ions which are co-doped in Co-Zn ferrites spinel structure by sintering, and a particular higher value of 0.96–0.97 in 8–14 μm emissivity is observed in the RE and Mn co-doped ferrites. Xiaoyan Wu et al. [21], developed the highest infrared emissivity (0.92 ± 0.01) and (0.95 ± 0.01) in 8–14 μm after doping Ce^3+^ and Y^3+^ from CoFe_2_O_4_ in which the maximum lattice strain (0.341%) and (4.40%) occurred after sintering at 600 ℃. Jian Zhang et al. [22], utilized transition metal ions (Cu^2+^, Co^3+^, Ni^2+^ and Zn^2+^) to dope and co-dope in Fe_3_O_4_ spinel ferrite by sintering process and the results show that the radiation emissivity in 3–5 μm and 8–14 μm can be increased above 0.9 at 600 ℃. These deferent emissivities are mainly determined by the chemical composition and crystal structure of the infrared radiation materials [23]. When the regular structure of the crystal is destroyed by doping with different radii of the ions, the level of the energy transition occurs locally and thus enables the development of infrared radiation [24,25]. In this sense, the infrared radiation coating is generally made as a composite material [26,27,28,29,30,31,32].

According to the different components involved, coatings are classified into: FeO-MnO_2_ series, Al2O_3_-SiO_2_ series, ZrO_2_ series, CoO series, CrO_2_ series, cordierite series and SiC series [29,33,34,35,36,37,38,39,40,41]. After conducting research on the different series, researchers have found that materials with spinel structures have higher infrared emissivity than other materials [42,43,44]. Although the theoretical basis for the application of infrared radiation has progressed to date, the composition of the coating still depends on experimentation. It is thus considered that effective methods and theories are required to design a formula for the coating that optimizes its infrared radiation performance. In this paper, a series of ${\mathrm{Cu}}_{x}{\mathrm{Co}}_{1-x}{\mathrm{Fe}}_{2}O_{4}$ solid solutions with a spinel structure was developed to effectively improve emissivity [22]. The ${\mathrm{Cu}}_{x}{\mathrm{Co}}_{1-x}{\mathrm{Fe}}_{2}O_{4}$ series material with infrared radiation coating was prepared in a laboratory, and the emissivity was then measured. According to the experimental results, we didn’t use mature mathematical prediction models. It should be noted that only the predicted results can be obtained by using these models, but it is not clear which factors affect them. We first determine the weight of the influencing factors, then establish the optimization model, and determine the best formula of spinel material and spinel-based infrared radiation coating.

## 2. Experimental

${\mathrm{Cu}}_{x}{\mathrm{Co}}_{1-x}{\mathrm{Fe}}_{2}O_{4}$ solid solution was selected for the study as it has a typical spinel structure, and Cu enables the formation of a mixed spinel structure during sintering [45]. The ${\mathrm{Cu}}_{x}{\mathrm{Co}}_{1-x}{\mathrm{Fe}}_{2}O_{4}$ were prepared by a sintering process. Analytical grade Co_2_O_3_ (99.99%), CuO (99.99%) and Fe_2_O_3_ (99.99%) powders were purchased from Shanghai Aladdin Bio-Chem Technology Co., LTD (Shanghai, China)) and used as raw materials to prepare ${\mathrm{Cu}}_{x}{\mathrm{Co}}_{1-x}{\mathrm{Fe}}_{2}O_{4}$. The varying formulas of ${\mathrm{Cu}}_{x}{\mathrm{Co}}_{1-x}{\mathrm{Fe}}_{2}O_{4}$ were designed according to different Cu doped ratios (mol%), and the molar content of CuO was regarded as being theoretically proportional to the content of the solid solution [22]. The formula design is shown in Table 1.

**Figure 1:**
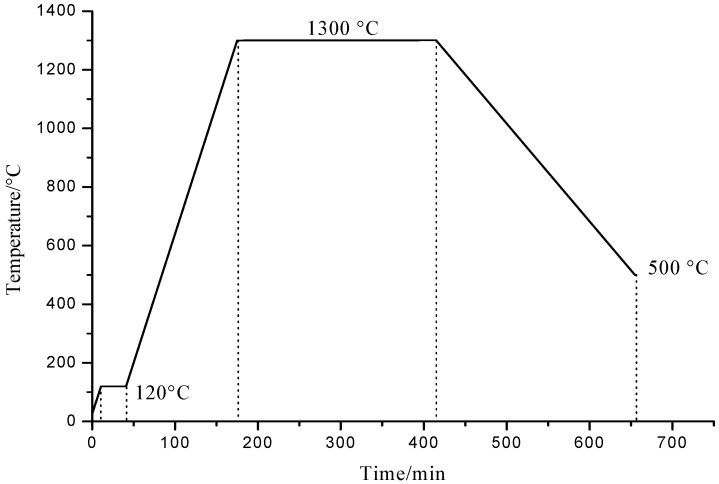
Temperature control curve of heat treatment.

Each component was weighed according to the formula weight design in Table 1 and mixed in an agate mortar, and samples were then heat treated under air atmosphere and sintered in a muffle furnace (Shanghai YongWei Furnace Industry Co. LTD, Shanghai, China), according to the temperature control curve of the heat treatment shown in Figure 1.

The samples were heated to 120 °C and kept for 30 min, so that they were fully dry. In order to ensure the reaction was as complete as possible, the samples were heated at 1300 °C for about 4 h and then cooled to 500 °C at a cooling speed of 5 °C/min. Finally, the samples were cooled naturally to room temperature in a muffle furnace. After the sintering process, the samples were ground to powder and those that could pass through a 200 mesh sieve were collected for further study.

In this study, the micromorphology of samples were analyzed after sintering using a scanning electron microscopy (SEM, Instrument model: ZEISS EVO18, Carl Zeiss AG, Heidenheim, Germany). An energy dispersive spectrometer (EDS, Carl Zeiss AG, Heidenheim, German) and an x-ray diffractometer (XRD, Instrument model: AXISULTRADLD, Kratos Analytical Limited, Kyoto, Japan) were used to verify the generation of the ${\mathrm{Cu}}_{x}{\mathrm{Co}}_{1-x}{\mathrm{Fe}}_{2}O_{4}$ solid solution. The XRD pattern was measured by X-ray diffractometer with the scan range angle from 10° to 90° at the speed of 10°/min. The emissivity of 3–5 and 8–14 μm band were measured by using a dual-band radiation emissivity measuring meter ((IR-2, Shanghai WangJia Optoelectronic Technology Co., LTD, Shanghai, China). In this paper, the emissivity of each sample in the 3–5 μm and 8–14 μm wavebands has been measured 25 times, and the average emissivity is given.

## 3. Results

### 3.1. Morphology of Samples

**Figure 2:**
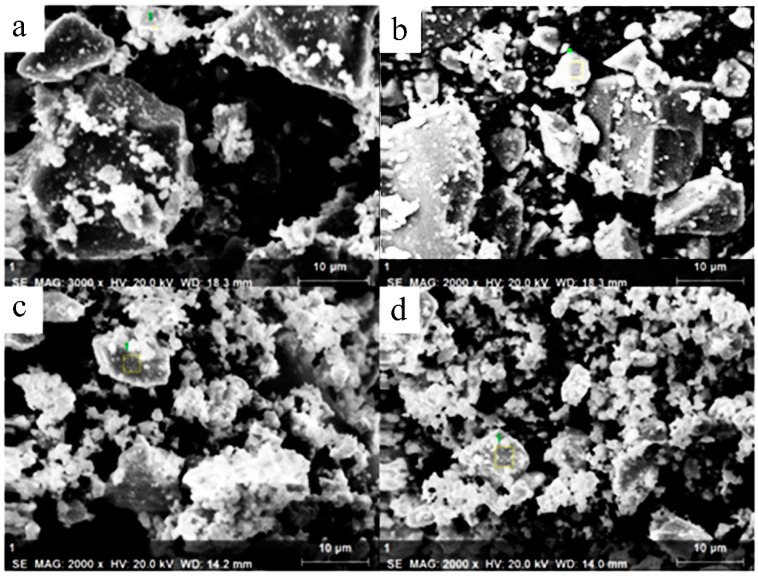
SEM images of samples S2 (**a**), S3 (**b**), S5 (**c**) and S7 (**d**).

The SEM images of test numbers S2, S3, S5 and S7 in Figure 2 show that the crystal particles of the spinel are irregular polyhedrons. The particles became more irregular with the increased addition of Cu, which may be the result of Cu doping destroying the polarized growth of the spinel crystal. In order to know whether the Cu had been dissolved in the spinel, energy dispersive spectrometer (EDS) analysis was carried out, and the results are shown in Table 2.

As can be seen from Table 2, the values of Cu/(Cu + Co) were 13.9%, 23.7%, 64.97% and 92.8%, which are consistent with the design value of Cu/Co. This illustrates that the Cu had been dissolved in the samples.

### 3.2. XRD Analysis

**Figure 3:**
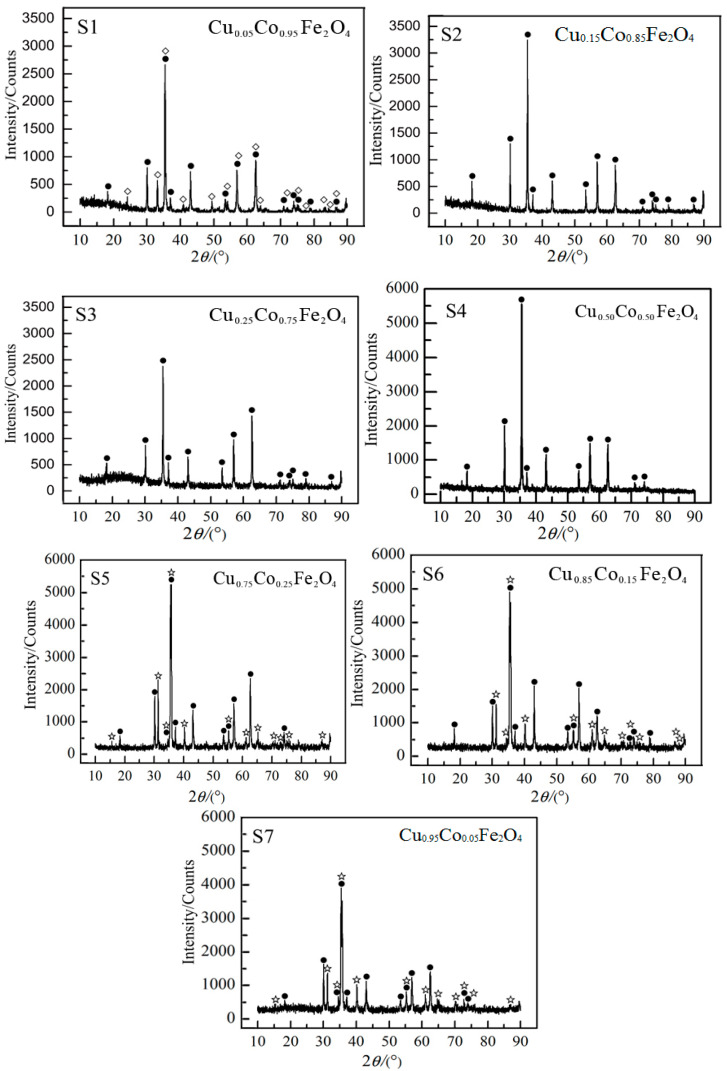
XRD pattern of the sample. The symbols •, ◇ and ☆ represent spinel, Fe_2_O_3_ and delafossite, respectively. Cu_0.05_Co_0.95_Fe_2_O_4_ (S1); Cu_0.15_Co_0.85_Fe_2_O_4_ (S2); Cu_0.25_Co_0.75_Fe_2_O_4_ (S3); Cu_0.50_Co_0.50_Fe_2_O_4_ (S4); Cu_0.85_Co_0.15_Fe_2_O_4_ (S5); Cu_0.95_Co_0.05_Fe_2_O_4_ (S6); Cu_0.75_Co_0.25_Fe_2_O_4_ (S7).

The XRD patterns of the samples shown in Figure 3 and we can observe characteristic peaks of spinel structure for samples are in accordance with the Joint Committee Powder Diffraction Standard (PDF# 22-1086). This indicates that the spinel structure was prepared. However, there existed other compositions like Fe_2_O_3_ and delafossite (CuFeO_2_), which are not conducive to the increase of emissivity because they are transparent to infrared radiation [16]. The reason for the presence of Fe_2_O_3_ in sample S1 is that improper stoichiometric ratio in the raw materials led to excessive Fe_2_O_3_ existed as remnant. On the other hand, in samples S5, S6 and S7, due to excessive CuO in the raw materials, undoped CuFe_2_O_4_ tended to be formed, which led to reaction (1) occurred when the temperature was above 1100 °C. Together with reactions (2) and (3) also under high temperature [46], delafossite (CuFeO_2_) could be formed in corresponding samples.

(1) $$4CuFe_{2}O_{4}\to 4CuFeO_{2}+2Fe_{2}O_{3}+O_{2}$$

(2) $$4CuO\to 2Cu_{2}O+O_{2}$$

(3) $$Cu_{2}O+Fe_{2}O_{3}\to 2CuFeO_{2}$$

In this sense, the two compositions should be avoided in the process of sintering to eliminating possible adverse effects on infrared radiation performance. Thus, it is necessary to arrange the formula with proper content of raw materials to obtain Cu_x_Co_1−x_Fe_2_O_4_ materials for excellent infrared radiation performance.

### 3.3. Strengthening Effect of Emissivity

**Figure 4:**
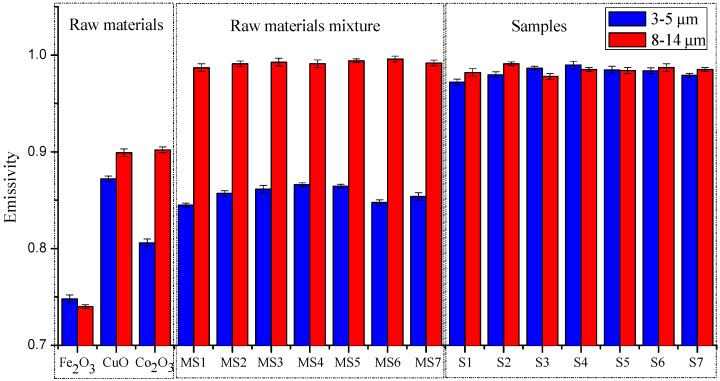
Emissivities of various formulas in 3–5 and 8–14 μm wavebands.

Owing to the complicated mechanism Cu_0.95_Co_0.05_Fe_2_O_4_ involved in the emissivity strengthening effect of a solid solution, it is difficult to ascertain whether there is a positive correlation between the emissivity strengthening and the degree of the solid solution. In order to clarify how the solid solution affects the emissivity, the emissivity of two wave ranges (3–5 μm and 8–14 μm) for the spinel-materials of ${\mathrm{Cu}}_{x}{\mathrm{Co}}_{1-x}{\mathrm{Fe}}_{2}O_{4}$ solid solution, based on different Cu doped ratios, were measured under 773 K (IR-2 dual band emissivity measurement instrument). Control experiments were conducted in order to validate the emissivity strengthening effect of the ferrite spinel solid solution, in which the emissivity of the chemicals CuO, Co_2_O_3_ and Fe_2_O_3_ were measured. The results are shown in Figure 4.

The emissivities of CuO, Co_2_O_3_ and Fe_2_O_3_ were measured, and the average values of emissivity were found to be 0.87, 0.81 and 0.75, respectively, in the 3–5 μm waveband at a temperature of 773 K. In the 8–14 μm waveband, the average values of emissivity were 0.90, 0.90 and 0.74 respectively. In Figure 4, the error bar of all the samples is under ±0.004 which means the accuracy of measured emissivity is believable. Furthermore, the mixing of the three oxides was found to make no contribution to the increase of emissivity in the 3–5 μm waveband, and there was no decrease in the emissivity of samples after sintering in the 8–14 μm waveband. However, samples S1–S7, which were sintered to be the ${\mathrm{Cu}}_{x}{\mathrm{Co}}_{1-x}{\mathrm{Fe}}_{2}O_{4}$ solid solution, were found to remarkably increase emissivity, particularly in the waveband of 3–5 μm. It was therefore clarified that the strengthening effect of the ferrite spinel solid solution was effective. 

However, although the emissivity seen in Figure 4 was high, the possibility of obtaining an optimal composition for the highest emissivity was considered. Thus, in order to obtain the best emissivity performance for the ${\mathrm{Cu}}_{x}{\mathrm{Co}}_{1-x}{\mathrm{Fe}}_{2}O_{4}$ solid solution, or for the coating in where the spinel material is the main composition, it was considered that optimized models, further calculations and analyses were required.

## 4. Optimization Analysis for Formula Design of Coating Slurry

This study proposes a novel optimization model design for the formula of ${\mathrm{Cu}}_{x}{\mathrm{Co}}_{1-x}{\mathrm{Fe}}_{2}O_{4}$ spinel-based coating slurry, with particular consideration for near and middle infrared radiation strengthening. To obtain the best result, the optimization included two steps. The first step was to optimize the ${\mathrm{Cu}}_{x}{\mathrm{Co}}_{1-x}{\mathrm{Fe}}_{2}O_{4}$ material, which is then used as the base in the further slurry formula. The second step was to optimize the coating slurry. Both formulas were optimized using the same model, and only the regressions used for the experimental data were different.

### 4.1. Optimization Model

The purpose of this research was to find an optimal high-emissivity coating for the surface of conventional refractory materials, such as high-aluminum bricks, castable refractories and clay bricks, which generally have low emissivity. It is of note that for widely used refractory materials, emissivity in the waveband of 1–5 μm is much lower than when the wavelength of radiation absorbed or emitted is bigger than 5 μm [47]. Therefore, if an infrared radiation coating with a higher near-infrared waveband emissivity is applied to the surface of the inner furnace, the radiation heat transfer will be strengthened and the furnace efficiency significantly improved [48,49,50,51]. Since the spinel material prepared in this study showed excellent performance in relation to its near-infrared radiation properties, it was therefore considered necessary to develop a high-performance spinel material ${\mathrm{Cu}}_{x}{\mathrm{Co}}_{1-x}{\mathrm{Fe}}_{2}O_{4}$ and corresponding coating slurry, using an optimization method that considers the near-infrared radiation property strengthening [52,53,54].

**Figure 5:**
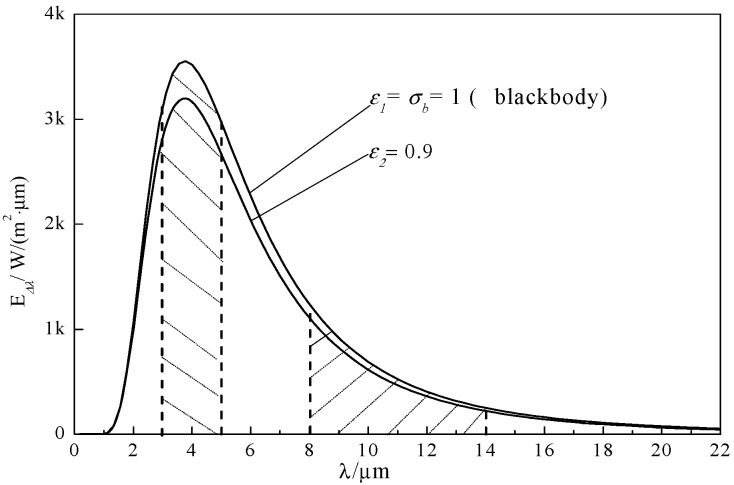
Radiation ability of blackbody and graybody at 773 K.

According to Planck’s law, the curve, E_λ_-λ, of a graybody’s surface with an emissivity of 0.9 can be obtained at 773 K, as shown in Figure 5. The area below the curve is divided into five parts and indicates the emissive power in the 1–22 μm waveband. The proportion of each part can be obtained using integral computing, and the result is shown in Table 3.

If the emissivity of each waveband is known, the weighted average emissivity in 1–22 μm can be expressed as follows, where the proportional value of each part is the weighting factor:

(4) $$\bar{\varepsilon }=0.13\varepsilon_{1}+0.34\varepsilon_{2}+0.30\varepsilon_{3}+0.18\varepsilon_{4}+0.05\varepsilon_{5}$$

For each sample that is based on different formulas, the emissivity, ε*_i_*, will be different in every waveband, leading to different values of $\bar{\varepsilon }$. The radiation ability of blackbody at 3–5 μm and 8–14 μm is the limit state of all high emissivity materials. This means that the radiation ability of materials is smaller than that of blackbody. At the same time, the emissivity (ε) is the ratio of E to $E_{b}$, where E is the radiation ability of materials and $E_{b}$ is the radiation ability of blackbody. In this respect, the radiation ability of Cu_x_Co_1−x_Fe_2_O_4_ spinel in 3–5 μm and 8–14 μm is closer to that of blackbody at 3–5 μm and 8–14 μm, the higher the emissivity is in 3–5 μm and 8–14 μm. It thus follows that there must be some relation between the values of $\bar{\varepsilon }$ and the contents of each formula. The optimization method was therefore designed to obtain the optimal formula by solving the extreme value problem for a maximum value of $\bar{\varepsilon }$. The dual-band radiation emissivity measuring meter was used for the measurement of emissivity in wavebands of 3–5 μm and 8–14 μm, and thus, Equation (5) was simplified as follows:

(5) $$\bar{\varepsilon }=0.34\varepsilon_{2}+0.18\varepsilon_{4}$$

### 4.2. Optimization of Cu_x_Co_1−x_Fe_2_O_4_ Material

The mole fraction of Cu, expressed as x, is the independent variable function of each waveband’s emissivity. Optimization of the formula was carried out according to the data shown in Figure 3, and the precision is able to meet the demand when the fitted equation is a six-order poly-nominal function. The results of fitting are shown as follows:

(6) $$\begin{array}{l} \varepsilon_{2}=-1.5083\times 10^{-11}x^{6}+4.8166\times 10^{-9}x^{5}-5.9452\times 10^{-7}x^{4}+3.5362\times 10^{-5}x^{3}\\ -1.0212\times 10^{-3}x^{2}+0.012457x+0.94118 \end{array}$$

(7) $$\begin{array}{l} \varepsilon_{4}=-4.069\times 10^{-13}x^{6}+2.4849\times 10^{-11}x^{5}+9.5818\times 10^{-9}x^{4}-1.1785\times 10^{-6}x^{3}\\ +3.3566\times 10^{-5}x^{2}+4.6237\times 10^{-4}x+0.96899 \end{array}$$

**Figure 6:**
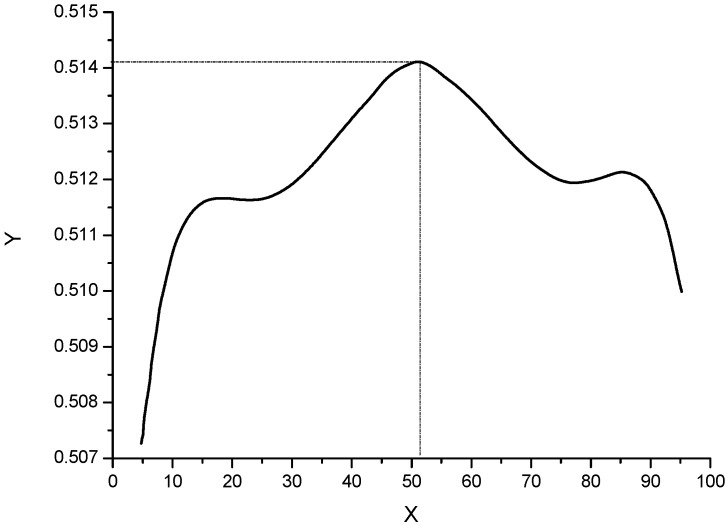
Curve of optimized objective function of Cu_x_Co_1−x_Fe_2_O_4_ materials.

Using the Matlab software, extreme computing with the objective function based on Equation (5) to obtain the effectual extreme point (the curve seen in Figure 6) delivered the mole fraction of Cu as 51.41%, and the corresponding optimal formula of spinal materials was calculated as shown in Table 4.

After preparing a sample based on the optimal formula, it was tested and the emissivity values are shown in Table 5, where the fitted values are also given for comparison. As shown in Table 5, the fitted and experimental results are in good agreement with each other.

### 4.3. Optimization of Coating Slurry

The spinel-based infrared radiation coating slurry is composed of ${\mathrm{Cu}}_{x}{\mathrm{Co}}_{1-x}{\mathrm{Fe}}_{2}O_{4}$ series material, with the addition of an adhesive, dispersant and thickener [55]. These additives are used to maintain the performance constant of the slurry, but they also affect the emissivity of the coating. Thus, to obtain the optimal formula for the coating slurry with respect to the optimized emissivity, it was considered necessary to study the relation between the components of the slurry and the emissivity.

Water glass (Na_2_SiO_3_ and silica), bentonite and sodium hexametaphosphate were selected as the adhesive, dispersant and thickener, respectively. The content of each component, expressed in mass percentage, was determined using a mixture regression design, which included the extreme vertices design, the boundary surface centroid design and the overall centroid design. If the number of factors is p in the mixture regression design, the content of the pth factor can be expressed as *x_p_*, and the criteria for the design can be concluded as follows:

(8) $$\{\begin{array}{c} 0\leq x_{i}\leq 1\;(i=1,2,3,\ldots ,p)\\ x_{1}+x_{2}+x_{3}+\ldots +x_{p}=1 \end{array}$$

In our design, the variation range of each component’s mass percentage, *x_i_*, was obtained based on consulting numerous references, and these are shown as follows:

(9) $$\{\begin{array}{cc} \;\mathrm{spinel}\;\mathrm{material}\;(x_{1}): & 0.3\leq x_{1}\leq 0.6\\ \mathrm{sodium}\;\mathrm{hexametaphosphate}\;(x_{2}): & 0.05\leq x_{2}\leq 0.15\\ \;\mathrm{bentonite}\;(x_{3}): & 0.15\leq x_{3}\leq 0.2\\ \;\mathrm{water}\;\mathrm{glass}\;(x_{4}): & 0.3\leq x_{4}\leq 0.5 \end{array}$$

Certain formulas have previously been obtained according to different methods and these are listed in Table 6.

According to Table 5, a total of 15 formulas were prepared in which there were different components in *x*_1_, *x*_2_, *x*_3_ and *x*_4_, and 15 types of coating slurry were then made according to the following procedure. Firstly, the adhesive and dispersant were mixed, the thickener was added and the high emissivity materials were then added and mixed. A certain amount of water and anhydrous ethanol were finally added, and after high-speed stirring the high emissivity coating slurry was produced. After the various slurries were coated on the surface of mullite refractory bricks and dried, the samples were then measured for emissivity.

The mathematical model (a quadratic form) used for the regression equation is shown in Equation (10), and it was selected for the mixture design in experiments:

(10) $$\hat{\varepsilon }=\sum_{i=1}^{p}b_{i}x_{i}+\sum_{i<j}b_{ij}x_{i}x_{j}$$

where *b_i_* and *b_ij_* are the factors to be obtained by regression of the experimental data. The infrared radiation coatings with four components make the *p* equal four, and thus, Equation (10) can be expressed as follows:

(11) $$\hat{\varepsilon }=\sum_{i=1}^{4}b_{i}x_{i}+\sum_{i<j}b_{ij}x_{i}x_{j}$$

Based on the mathematic model and the data of emissivity of each sample (as shown in Table 7), the factors *b_i_* and *b_ij_* in Equation (11) can be defined as the regression of the experiment using SPSS statistical software. In Table 7, *y*^1^ and *y*^2^ mean that the emissivity of samples in 3–5 μm and 8–14 μm band, respectively.

Based on the data in Table 7, the regression equations were obtained using SPSS statistical software, and the regression equations of 3–5 µm and 8–14 µm can thus be expressed as Equation (12) and Equation (13), respectively:

(12) $$\begin{array}{l} \varepsilon_{2}=-8.574x_{2}+0.087x_{3}-2.329x_{4}+5.234x_{1}x_{2}-7.255x_{1}x_{3}+1.395x_{1}x_{4}\\ +11.385x_{2}x_{3}+13.991x_{2}x_{4}+2.065x_{3}x_{4}+1.858 \end{array}$$

(13) $$\begin{array}{l} \varepsilon_{4}=5.892x_{2}+6.393x_{3}+0.031x_{4}-6.477x_{1}x_{2}-11.1x_{1}x_{3}+2.738x_{1}x_{4}\\ -11.828x_{2}x_{3}-9.493x_{2}x_{4}-8.49x_{3}x_{4}+0.849 \end{array}$$

The same method was used in the ${\mathrm{Cu}}_{x}{\mathrm{Co}}_{1-x}{\mathrm{Fe}}_{2}O_{4}$ series material optimization, and Equations (12) and (13) can be combined into the objective function, Equation (5). The final regression equation is shown as follows:

(14) $$\begin{array}{l} \varepsilon =0.34\varepsilon_{2}+0.18\varepsilon_{4}=-1.8546x_{2}+1.18032x_{3}-0.78628x_{4}+0.6137x_{1}x_{2}\\ -4.4647x_{1}x_{3}+0.96714x_{1}x_{4}+1.74186x_{2}x_{3}+3.0482x_{2}x_{4}-0.8261x_{3}x_{4}+0.784 \end{array}$$

The extremum of the equations in the ranges of mass fractions were computed using Matlab software, and the results were: *x*_1_ = 30%, *x*_2_ = 14.16%, *x*_3_ = 20% and *x*_4_ = 35.84%. Thus, the emissivities for the 3–5 μm and 8–14 μm wavebands were obtained as 0.944 and 0.901, respectively.

In order to verify the accuracy of the model, the coating was prepared according to the optimal formula, and the emissivity was measured at 773 K. The fitting values and experimental values are shown in Table 8.

Deviations of the fitting values from the experimental values in both the 3–5 μm and 8–14 μm wavebands, were 1.4% and 0.44% respectively, which implies the model is capable of reflecting the relation between emissivity and the components of the slurry.

In summary, the emissivities of the optimal formulas for the ${\mathrm{Cu}}_{x}{\mathrm{Co}}_{1-x}{\mathrm{Fe}}_{2}O_{4}$ series material and coatings were compared in both the 3–5 μm and 8–14 μm wavebands and are shown in Table 9.

As can be seen from Table 9, the emissivity of the coating was slightly lower than the emissivity of the materials, but all values were more than 0.9 in both wavebands. In addition, the coating has a good radiation performance in the 3–5 μm waveband at 773 K, which illustrates that the preparation of the coating was successful.

## 5. Conclusions

In this study, a ${\mathrm{Cu}}_{x}{\mathrm{Co}}_{1-x}{\mathrm{Fe}}_{2}O_{4}$ series infrared radiation material with a spinel structure was successfully prepared, and using the ${\mathrm{Cu}}_{x}{\mathrm{Co}}_{1-x}{\mathrm{Fe}}_{2}O_{4}$ series material, an adhesive, dispersant and thickener as basic components, 15 formulas were designed for an infrared radiation coating slurry. The coating slurry was then prepared and the emissivity was measured. A model was then constructed and applied to optimize the formulas of both the ${\mathrm{Cu}}_{x}{\mathrm{Co}}_{1-x}{\mathrm{Fe}}_{2}O_{4}$ series material and its coating slurry, and verification tests were conducted. Conclusions were obtained as follows:

(1) The optimal formula for the ${\mathrm{Cu}}_{x}{\mathrm{Co}}_{1-x}{\mathrm{Fe}}_{2}O_{4}$ series infrared radiation material was CuO 16.98%, Co_2_O_3_ 16.73% and Fe_2_O_3_ 66.29%. At 773 K, the experimental emissivities of the formula were 0.986 and 0.977 in the 3–5 μm and 8–14 μm waveband, respectively.
(2) Based on the ${\mathrm{Cu}}_{x}{\mathrm{Co}}_{1-x}{\mathrm{Fe}}_{2}O_{4}$ series material, a spinel high emissivity coating was prepared and its performance analyzed. In addition, the optimized formula for high emissivity was obtained using the model with Matlab software. The optimal formula obtained was as follows: binder 30%; sodium hexametaphosphate 14.16%; bentonite 20% and water glass 35.84%. At 773 K, the emissivities of the formula were calculated as 0.931 and 0.905 in the 3–5 μm waveband and 8–14 μm waveband, respectively, which are close to the corresponding measured values.
(3) In this study, the emissivity of the semiconductor with a spinel structure, such as the ${\mathrm{Cu}}_{x}{\mathrm{Co}}_{1-x}{\mathrm{Fe}}_{2}O_{4}$ solid solution, in the near and middle infrared wavebands was a key consideration, and this was applied to the optimized process using the model, and as such is confirmed in the experiments.

## Figures and Tables

**Table 1:** Formula for Cu_x_Co_1−x_Fe_2_O_4_ solid solution.

Test Number	Formula/g			Cu Doped Ratio/%
	CuO	Co_2_O_3_	Fe_2_O_3_	
S1	1.6925	33.5258	67.9524	5
S2	5.0774	29.9968	67.9524	15
S3	8.4623	26.4678	67.9524	25
S4	16.9246	17.6452	67.9524	50
S5	25.3868	8.8226	67.9524	75
S6	28.7717	5.2936	67.9524	85
S7	32.1567	1.7645	67.9524	95

**Table 2:** EDS result of samples S2, S3, S5 and S7 and the mole ratios of Cu/(Cu + Co).

Samples	The Mole Fraction of Each Element/%				Cu/(Cu + Co)/%
	O	Fe	Cu	Co	
S2	58.12	27.28	2.04	12.57	13.9
S3	55.30	30.02	3.48	11.20	23.7
S5	56.24	31.19	8.16	4.40	64.97
S7	45.81	37.02	15.96	1.22	92.8

**Table 3:** Proportion of each waveband for blackbody at 773 K.

Waveband	1–3 μm	3–5 μm	5–8 μm	8–14 μm	14–22 μm
Proportion	0.13	0.34	0.30	0.18	0.05

**Table 4:** Optimal formula of spinel materials.

Composition	CuO	Co_2_O_3_	Fe_2_O_3_
Mass Content/%	16.98	16.73	66.29

**Table 5:** Experimental values and fitted values of spinel materials.

Waveband	3–5 μm	8–14 μm
Fitted values	0.99	0.986
Experimental values	0.986	0.977
Deviation	0.41%	0.92%

**Table 6:** Design formula using different design methods.

Number		*x*_1_	*x*_2_	*x*_3_	*x*_4_
Extreme Vertices Design	1	0.50	0.05	0.15	0.30
	2	0.30	0.05	0.15	0.50
	3	0.45	0.05	0.20	0.30
	4	0.40	0.15	0.15	0.30
	5	0.35	0.15	0.20	0.30
	6	0.30	0.05	0.20	0.45
	7	0.30	0.15	0.15	0.40
	8	0.30	0.15	0.20	0.35
Boundary Surface Centroid Design	9	0.36	0.10	0.18	0.36
	10	0.30	0.10	0.18	0.42
	11	0.39	0.05	0.18	0.38
	12	0.34	0.15	0.18	0.33
	13	0.38	0.10	0.15	0.37
	14	0.35	0.10	0.20	0.35
Overall Centroid Design	15	0.42	0.10	0.18	0.30

**Table 7:** Emissivity of each formula.

Number	*x*_1_	*x*_2_	*x*_3_	*x*_4_	*y*_1_	*y*_2_
1	0.50	0.05	0.15	0.30	0.928	0.914
2	0.30	0.05	0.15	0.50	0.830	0.970
3	0.45	0.05	0.20	0.30	0.849	0.887
4	0.40	0.15	0.15	0.30	0.912	0.900
5	0.35	0.15	0.20	0.30	0.900	0.900
6	0.30	0.05	0.20	0.45	0.845	0.947
7	0.30	0.15	0.15	0.40	0.950	0.897
8	0.30	0.15	0.15	0.40	0.943	0.905
9	0.36	0.10	0.18	0.36	0.926	0.903
10	0.30	0.10	0.18	0.42	0.927	0.899
11	0.39	0.05	0.18	0.38	0.867	0.939
12	0.34	0.15	0.18	0.33	0.923	0.894
13	0.38	0.10	0.15	0.37	0.935	0.913
14	0.35	0.10	0.20	0.35	0.910	0.894
15	0.42	0.10	0.18	0.30	0.905	0.876

**Table 8:** Fitting values and experimental values.

Waveband	3–5 μm	8–14 μm
Fitting values	0.944	0.901
Experimental values	0.931	0.905
Deviation	1.4%	0.44%

**Table 9:** Emissivities of Cu_x_Co_1−x_Fe_2_O_4_ series infrared radiation material and coating.

Waveband	3–5 μm	8–14 μm
Optimal materials	0.986	0.977
Optimal coating	0.931	0.905
